# Noninvasive assessment of steatosis and viability of cold-stored human liver grafts by MRI

**DOI:** 10.1002/mrm.28930

**Published:** 2021-07-17

**Authors:** Liam A. J. Young, Carlo D. L. Ceresa, Ferenc E. Mózes, Jane Ellis, Ladislav Valkovič, Richard Colling, Constantin-C. Coussios, Peter J. Friend, Christopher T. Rodgers

**Affiliations:** 1 Oxford Centre for Clinical Magnetic Resonance Research, Radcliffe Department of Medicine, University of Oxford, Oxford, United Kingdom; 2 Nuffield Department of Surgical Sciences, University of Oxford, Oxford, United Kingdom; 3 Department of Imaging Methods, Institute of Measurement Science, Slovak Academy of Sciences, Bratislava, Slovakia; 4 institute of Biomedical Engineering, University of Oxford, Oxford, United Kingdom; 5 Wolfson Brain Imaging Centre, Department of Clinical Neurosciences, University of Cambridge, Cambridge, United Kingdom

**Keywords:** liver transplantation, MOLLI T_1_, normothermic machine perfusion, proton density fat fraction, static cold storage, temperature sensitivity

## Abstract

**Purpose:**

A shortage of suitable donor livers is driving increased use of higher risk livers for transplantation. However, current biomarkers are not sensitive and specific enough to predict posttransplant liver function. This is limiting the expansion of the donor pool. Therefore, better noninvasive tests are required to determine which livers will function following implantation and hence can be safely transplanted. This study assesses the temperature sensitivity of proton density fat fraction and relaxometry parameters and examines their potential for assessment of liver function ex vivo.

**Methods:**

Six ex vivo human livers were scanned during static cold storage following normothermic machine perfusion. Proton density fat fraction, T_1_, T_2_, and ${\text{T}}_{2}^{*}$ were measured repeatedly during cooling on ice. Temperature corrections were derived from these measurements for the parameters that showed significant variation with temperature.

**Results:**

Strong linear temperature sensitivities were observed for proton density fat fraction (R^2^ = 0.61, *P* < .001) and T_1_ (R^2^ = 0.78, *P* < .001). Temperature correction according to a linear model reduced the coefficient of repeatability in these measurements by 41% and 36%, respectively. No temperature dependence was observed in T_2_ or ${\text{T}}_{2}^{*}$ measurements. Comparing livers deemed functional and nonfunctional during normothermic machine perfusion by hemodynamic and biochemical criteria, T_1_ differed significantly: 516 ± 50 ms for functional versus 679 ± 60 ms for non-functional, *P* = .02.

**Conclusion:**

Temperature correction is essential for robust measurement of proton density fat fraction and T_1_ in cold-stored human livers. These parameters may provide a noninvasive measure of viability for transplantation.

## 1 Introduction

Liver transplantation is the only definitive treatment for end-stage liver disease. Annually, over 15 thousand liver transplants are performed in Europe and the United States.^1,2^ However, a shortage of suitable donor organs means that currently about 30% of patients will become too ill to receive a transplant or die before receiving a liver transplant.^2^ To counter this organ shortage, suboptimal, extended criteria donor livers are increasingly being used. However, these more “marginal” extended criteria donor organs carry a higher risk of posttransplant complications, including graft failure and patient mortality.^3^ Being able to predict a liver’s viability to survive the transplant process accurately will increase the number of extended criteria donor grafts that can be safely utilised. The major factors that have been identified as independent predictors of graft failure, patient mortality, and primary nonfunction posttransplantation include: increasing donor age,^4^ increasing cold ischemia time,^5^ whether the liver is donated following circulatory death rather than being donated following brain-stem death,^6^ and the presence of macrovesicular steatosis.^7^

The degree of macrovesicular steatosis is initially assessed by inspection of the macroscopic appearance of the liver by the implanting surgeon. However, this visual inspection is highly subjective and can misclassify the presence or extent of steatosis in up to 66% of livers.^7^ Some centers confirm macrovesicular steatosis levels with a liver biopsy before making a decision about transplantability. However, biopsy suffers from a large sampling error due to the small section of the liver examined and an inconsistent observer bias.^8^

A recent study by Mergental et al. demonstrated that through a combination of enhanced organ preservation with normothermic machine perfusion (NMP) and functional assessment of the graft prior to transplant, over 70% of donated livers previously deemed unsuitable for transplantation by the current criteria were able to be transplanted with good immediate outcomes.^9^ However, the perfusion dynamics and metabolic parameters used to evaluate livers in this study were not sufficient alone to identify all livers that developed later complications. New noninvasive biomarkers to assess the viability of a graft for transplantation have the potential to increase the range of usable extended criteria donor livers further still. They could identify which livers should be transplanted directly, which would benefit from enhanced preservation techniques, and which should be discarded.

MRI and MRS are noninvasive, nonionizing imaging techniques. In vivo, MRI and MRS are capable of accurately quantifying liver steatosis (eg, in terms of the proton density fat fraction [PDFF])^10^ and predicting clinical outcomes in a range of liver diseases (eg, using T_1_ mapping).^11,12^ The signal detected by MR methods is intrinsically a function of the net tissue magnetisation, which is described by the Boltzmann distribution, and the tissue relaxation rates, which are pre-dominantly determined by the rate of molecular tumbling. Magnetisation and relaxation both vary with temperature, and hence many MR parameters depend on temperature as well. During in vivo scans, it is usually assumed that the body is at a constant physiological temperature of 37°C. However, the current gold-standard preservation technique for livers following retrieval is static cold storage (SCS), where the liver is flushed with a cold preservation solution and placed in an ice bath to rapidly decrease its temperature. In other situations when the specimen temperature can change, for example, in phantoms,^13^ preclinical studies,^14^ or cadavers during post-mortem examinations,^15^ large variations in relaxation rates can be observed. Characterization and correction of relaxometry changes have been performed on in vivo livers during post-mortem scans,^15^ and correction of steatosis measurements with PDFF has been possible in ex vivo livers immersed in room temperature phosphate-buffered saline.^16^ However, little is known about the effect of temperature on relaxometry parameters and fat fraction measurements of donated human transplant livers in the temperature range typically seen during SCS (0-15°C^17^).

Therefore, we set out to examine the effect of temperature changes during SCS on relaxation and PDFF values, and to derive temperature corrections for relaxation and PDFF parameters with significant temperature sensitivities to enable accurate noninvasive assessment of ex vivo livers prior to transplantation irrespective of their temperature at the time of scanning. We further aimed to compare a range of spectroscopic and imaging techniques, assessing their potential for predicting an organ’s viability for transplantation through correlation with histopathology and with liver function during perfusion.

## 2 Methods

### 2.1 Study protocol

**Figure 1:**
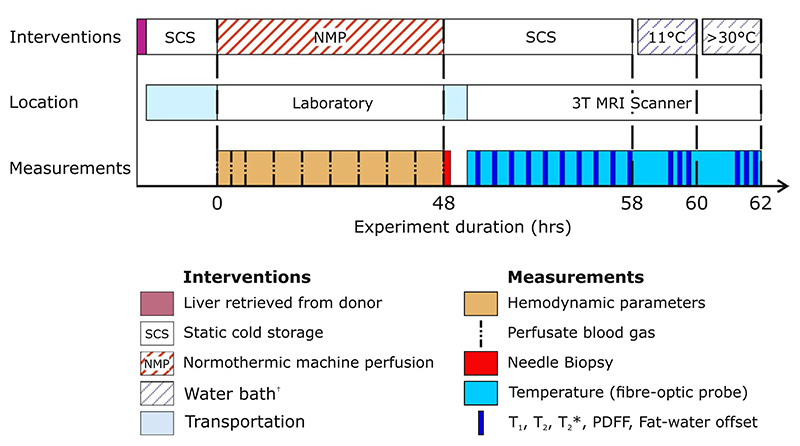
Timing of the study procedures for an ex vivo human liver. Liver function was assessed through regular arterial blood gas analysis and continuous monitoring of bile production and hemodynamic parameters as described in the study protocol. Throughout the MR examination T_1_, T_2_, T_2_, ${\text{T}}_{2}^{*}$ PDFF, and fat–water offset were all measured hourly on average. Invasive temperature measurements were made using a fiber-optic temperature probe in 3 livers; the remaining livers remained sealed inside sterile packaging throughout the study. Two livers were re-warmed in a water bath to differentiate the impact of storage duration and temperature on relaxometry and PDFF measures. Abbreviation: PDFF, proton density fat fraction

The study protocol is outlined in Figure 1. In brief, human livers that had been donated but deemed unsuitable for transplantation underwent 48 h of functional assessment during normothermic machine perfusion. A needle biopsy was taken at the end of perfusion for histopathology analysis. The livers were then cold flushed and placed on ice for a 10-h period of cold storage. During this time, T_1_, T_2_, ${\text{T}}_{2}^{*}$; fat–water frequency offset; and PDFF were measured hourly on average resulting in a total of 10 measurement time points per liver. A subset of livers (n = 2) were then re-warmed in a water bath, and further relaxometry and PDFF measurements were made.

### 2.2 Liver specimens and preparation for MRI

Six human livers were retrieved for transplantation as part of a standard multi-organ deceased donor organ retrieval^18^ but were deemed unsuitable for transplantation due to the retrieval surgeon reporting the presence of severe steatosis. The livers were transported on ice as per standard practice before being cannulated and perfused on a clinical NMP device (metra, OrganOx, Oxford, UK) as described previously^19^ for 48 h. A lipoprotein apheresis filter (DALI 500, Fresenius Medical Care (UK) Ltd, Huthwaite, UK) was added to the perfusion circuit for all perfusions, and livers 2 and 3 were supplemented with de-fatting interventions of: 1 g l-carnitine hydrochloride (Sigma-Aldrich, Gillingham, UK), 1 mg NKH477 hydrochloride (Cayman Chemical, Ann Arbor, MI), a reduced insulin level of 100 units (Actrapid, Novo Nordisk, Gatwick, UK), and a reduced perfusate glucose concentration of 5 mmol/L before total parenteral nutrition (Nutriflex Special, B Braun Medical Ltd, Sheffield, UK). At the end of perfusion, livers were cold-flushed with 3L histidine-tryptophan-ketoglutarate (HTK) solution (Custodiol, Pharmapal Ltd, Elstree, UK) at 4°C. Core biopsy samples were taken from the right lobe and fixed in 10% formalin. All livers were then placed into sealed bags with excess HTK solution and stored on ice. The study was approved by National Ethics Review Committee of the United Kingdom (REC reference 16/NE/0248).

### 2.3 MR examination

All livers underwent a 10-h MRI acquisition on a clinical 3 T system (Magnetom Tim Trio, Siemens Healthineers, Erlangen, Germany) fitted with a 32-channel ^1^H array coil (Rapid Biomedical, Rimpar, Germany) while inside an ice box. For all livers, scanning started less than 30 min after the end of perfusion. At the end of the protocol, 2 of the livers were immersed in a water bath at 11°C and scanned for 90 min before being placed into a warm water bath (at 30°C and 35°C, respectively) and scanned for an additional 90 min. All livers remained immersed in HTK solution and inside the sealed bags throughout the MRI procedure.

#### 2.3.1 MRS acquisition and analysis

To enable noninvasive monitoring of temperature, single voxel STEAM^20^ spectroscopy was performed in 4 voxels across the liver, with and without water suppression. Three voxels were placed in the right lobe, and one voxel was placed in the left, with all voxels positioned in parenchyma away from any obvious vasculature. Sequence parameters were: TE = 10 ms, mixing time = 7 ms, 20 × 20 × 20 mm^3^ voxel size, 16 averages with water suppression and 3 without, and TR = 750 ms with water suppression and TR = 4750 ms without. All spectra were quantified using the OXSA toolbox (version 2.0)^21^ in MatLab (MathWorks, Natick, MA). Spectra acquired with water suppression were used to quantify the lipid signals that are an order of magnitude lower than the water signal. The spectra without water suppression allowed quantification of water as a reference. The temperature dependent fat–water frequency shift^22^ was calculated as the difference between the main fat peak (methylene) and water. An average over all 4 liver voxels was calculated for global temperature assessment. To generate a linear calibration curve between fat–water frequency shift and temperature, a fibre optic temperature probe (Neoptix, Quebec City, Quebec, Canada) was inserted into the center of the right lobe of 3 livers and used to record actual temperature throughout the study.

An inversion recovery single voxel STEAM^20^ (STEAM-IR) sequence was also used to characterise the T_1_ of water in a voxel positioned near the center of the right lobe away from any obvious vasculature. Spectroscopy scans were repeated with TI = 50, 500, 1218, 2385, 3553, 4750, 5500, 7000 ms; TR = 10 s; 4 averages; 4 kHz bandwidth; and 1024 points. Additionally, a multi-TE, multi-TR STEAM single-voxel sequence was used to calculate a spectroscopic PDFF in the same voxel position.^23^ Parameters used for this sequence were: no water suppression; TR = 150, 175, 200, 225, 250, 275, 300, 325, 350, 400, 450, 500, 600, 700, 800, 900, 1000, 1250, 1500, 2000 ms, with a fixed TE of 15 ms before 8 spectra with TR = 1000 ms and TE = 20, 25, 30, 35, 50, 70, 90 and 110 ms. The 3 largest fat peaks were included in the prior knowledge, and a correction for the smaller peaks was performed using a previously described standard liver spectrum (see Supporting Information Table S1 for prior knowledge).^24^

The amplitudes of the water peak from inversion recovery data were fitted to:

(1) $$S_{i}=S_{0}(1\;-\;\exp(-\frac{TI_{i}}{T_{1w}})),$$

where *S*_0_ is a scaling factor proportional to proton density; *S*_i_ is the peak amplitude of a spectrum with *TI*_*i*_; and *T*_1*w*_ is the water *T*_1_. Peak amplitudes from the multiple TR and TE spectra were fitted to:

(2) $$S_{i}=S_{0}(1\;-\;\exp(-\frac{\tau }{T_{1}}))\;\exp(-\frac{TE}{T_{2}}),$$

where *S*_0_ is a scaling factor proportional to proton density and *τ* is the time from the final pulse in the STEAM sequence to the end of the TR interval.

The spectroscopic PDFF was defined as the ratio of corrected fat spectrum peak amplitudes to the sum of water and fat spectrum peak amplitudes, as shown in Equation (3).^25^


(3) $$PDFF[\%]=\frac{\sum_{i}S_{f_{i}}}{S_{w}+\sum_{i}S_{f_{i}}}\times 100$$

Here, *S*_*w*_ is the water peak amplitude, and *S*_*f*__*i*_ denotes the peak amplitude of the *i*^th^ fat peak.

#### 2.3.2 MRI acquisition and analysis

A shortened MOLLI (ShMOLLI) sequence was used to acquire coronal T_1_ maps. The ShMOLLI parameters were based on a standardized protocol (Siemens WIP 561a): TR/TE = 2.52/1.05 ms, 35° readout flip angle, 898 Hz/px band-width, 8 mm slice thickness, 7 TI, 110 ms first TI, 80 ms TI increment, 288 × 384 FOV, 192 × 182 matrix, GRAPPA acceleration factor R = 2, 24 reference lines, 6/8 partial Fourier in the phase encoding direction, and a simulated heart rate of 75 beats/min. T_1_ values were calculated using the ShMOLLI conditional fitting algorithm.^26^

A set of multiple-echo spoiled gradient recalled echo images was collected to obtain PDFF and ${\text{T}}_{2}^{*}$ maps. Parameters for the 2D multiple-echo gradient recalled echo sequence were: 6° flip angle; TR/TE = 500/ 1.25, 2.46, 3.69, 4.92, 6.15, 7.38, 8.61, 9.84 ms; 400 × 325 mm^2^ FOV; 6 mm slice thickness; 128 × 128 acquisition matrix; 1090 Hz/px bandwidth; and a bipolar gradient readout scheme. PDFF was calculated using the mixed-magnitude/complex-fitting method^27^ initialized with the graph cut method^28^ from the ISMRM Fat-Water Toolbox.^29^ Effects of the bipolar gradients were minimized by a high bandwidth, a linear phase correction,^30^ and use of the mixed-magnitude/complex-fitting method.^27^ Temperature correction of the modelled fat spectrum was performed by using the temperature estimate from the fat–water frequency shift measurements to adjust the fat offset frequency by 0.01 ppm/C, as proposed by Hernando et al.^13^ MRI PDFF was defined as the ratio of fat and water image intensities.

ROIs from both PDFF and T_1_ maps were extracted for the same voxel positions as the STEAM-IR and multi-TR multi-TE spectroscopy.

### 2.4 Functional assessment of livers during normothermic machine preservation

During NMP, hemodynamic parameters were monitored continuously, and perfusate blood gas analysis (ALB90 Flex, Radiometer, Crawley, UK) was performed at least once every 6 h throughout perfusion. At the end of perfusion and prior to MR examination, these parameters were used to determine functional viability of the liver according to recently published criteria.^31^ These criteria use perfusate lactate clearance as the primary biomarker. For a liver to be considered functional at the end of perfusion, it must metabolize perfusate lactate, bringing levels to ≤ 2.5 mmol/L within 4 h of perfusion onset and maintain the perfusate lactate concentration below ≤ 2.5 mmol/L for the remainder of the perfusion. Livers that clear lactate must also fulfil at least 2 of the following additional criteria to be considered functional: maintenance of stable portal vein and arterial flow rates of ≥ 500 and ≥ 150 mL/min, respectively; evidence of glucose metabolism; production of bile; maintenance of perfusate pH ≥ 7.30; and a visibly homogeneous perfusion with soft consistency of the parenchyma. Livers deemed both functional and nonfunctional were included in the MR analysis to determine whether MR parameters can differentiate between them.

### 2.5 Histological assessment

At the end of perfusion, core biopsy samples were taken from the edge of the right lobe using an 18G biopsy needle (Biopince Full Core Biopsy Instrument, Argon Medical Devices, Frisco, TX). Samples were immediately fixed in 10% formalin and later embedded in paraffin, cut into 4 μm sections, and stained with hematoxylin and eosin, periodic acid Schiff, and Sirius Red. A blinded pathologist graded the sections according to the semi-quantitative nonalcoholic fatty liver disease activity score (NAS; summation of steatosis 0-3, lobular inflammation 0-3, and hepatocyte ballooning 0-2).^32^ Additionally, macrovesicular steatosis was quantified on the hematoxylin and eosin stained sections by digital image analysis (Visiopharm application 10119, H&E liver steatosis, Visiopharm Ltd, Egham, UK).

### 2.6 Statistical analysis

A linear mixed-effects regression analysis was performed to assess the association of temperature and the MR parameters (T_1_ and fat fraction) while accounting for interdonor variability. Temperature was modelled as a fixed effect, assuming variation of T_1_ (or fat fraction) as a function of temperature is a constant between livers. The underlying temperature-independent T_1_ (or fat fraction) component for each liver was modelled as a random-effect component. Perturbations in the measurements due to temperatures were studied using liver-independent Δ*T*_1_ and ΔPDFF by subtraction of the temperature-independent component (a constant for each liver) from the MR parameters. Temperature-corrected MR parameters *T*_1_(0°C) and *PDFF*(0°C) were calculated for all measurements using the temperature fixed effect component and the calculated noninvasive temperature. The correlation of each MR parameter with temperature was evaluated using Pearson’s correlation coefficient (*ρ*), and the linearity of the correlation was assessed using the coefficient of determination (R^2^). For each MR parameter, the overall coefficient of variation (*CV*) was defined as the mean of the coefficients of variation calculated for each liver (*CV*_*i*_ = *σ_i_*/*μ_i_*, where *σ_i_* is the SD of all measurements performed in liver *i* and *μ_i_* is the mean of all measurements for liver *i*).^33^ Similarly, the coefficient of repeatability (*CR*) was defined as the mean of the coefficients of repeatability for each liver ($CR_{i}=1.96\times \sqrt{2}\times \sigma_{i}$).^34^ Unless otherwise stated, all measured results are presented as mean ± SD, and all fitting results are presented as mean value followed by 95% confidence intervals in brackets. A Student *t* test was used to determine statistical significance of differences in MR parameters between functional and nonfunctional livers. Where statistically significant differences were identified, potential cutoff values for the diagnosis of non-function were proposed such that sensitivity and specificity were > 80% and the Youden index was maximized.^35^ All statistical hypothesis testing was performed at significance level α = 0.05.

## 3 Results

### 3.1 Liver function during normothermic machine perfusion

Table 1 details the donor characteristics and the liver’s functional parameters during normothermic machine perfusion. Five livers initially cleared lactate in the first 4 h of perfusion, but only 3 livers maintained sufficient function to meet the functional viability criteria at the end of perfusion. Normothermic perfusion of liver 5 was terminated after just 36 h due to increasing perfusate acidosis, visually poor perfusion of the tissue, and increasing lactate levels that did not change following a lactate challenge. These factors meant the liver did not meet the functional viability criteria, and its function was so poor that it was deemed futile to continue perfusion to 48 h.

### 3.2 Noninvasive temperature measurement

**Figure 2:**
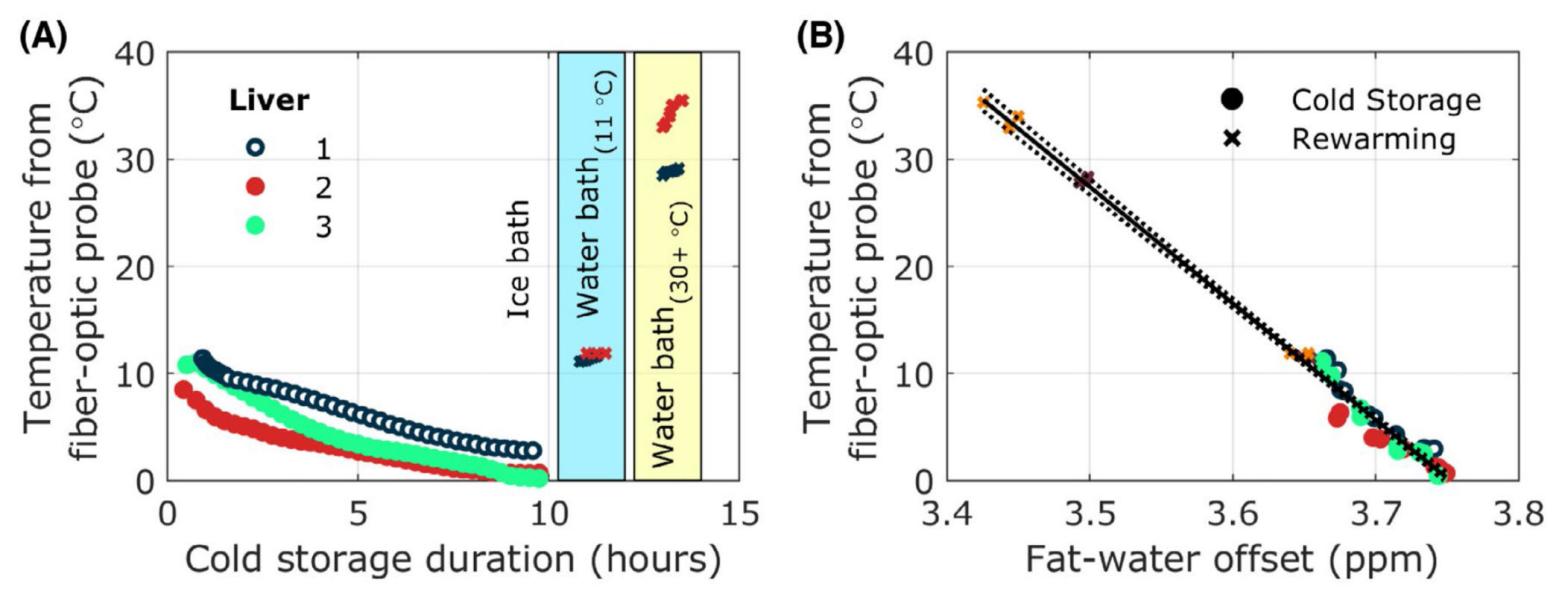
(A) Temperature changes in 3 discarded human livers during static cold storage and re-warming (of 2 livers) in a water bath measured using a fibre optic temperature probe in the right lobe. (B) Variation of chemical shift offset between the water and main fat (CH_2_) peak for the same 3 discarded human livers as a function of temperature measured using a fiber optic temperature probe in the right lobe

Figure 2A shows the temperature changes in the 3 livers fitted with fibre-optic temperature probes. The strong linear dependence of fat–water frequency offset calculated from single-TE MRS with temperature in the 3 livers is shown in Figure 2B (R^2^ = 0.988, *P* < .0001). The temperature sensitivity of the fat–water frequency offset was –0.0092 ± 0.0003 ppm/°C. Using this value as a conversion factor, an average decrease in temperature of 9.2 ± 4.3°C (range = 4.5-15.6°C) was noninvasively observed using the fat–water frequency offset in all livers during the 10 h of cold storage. Livers equilibrated to 1.4 ± 1.2°C after 10 h of cold storage. Comparing to earlier in the study, after the first 30 min of SCS liver temperatures were 10.6 ± 5.2°C, which was warmer and more variable than at 10 h, as would be expected from thermal cooling physics.

### 3.3 Relaxometry

**Figure 3:**
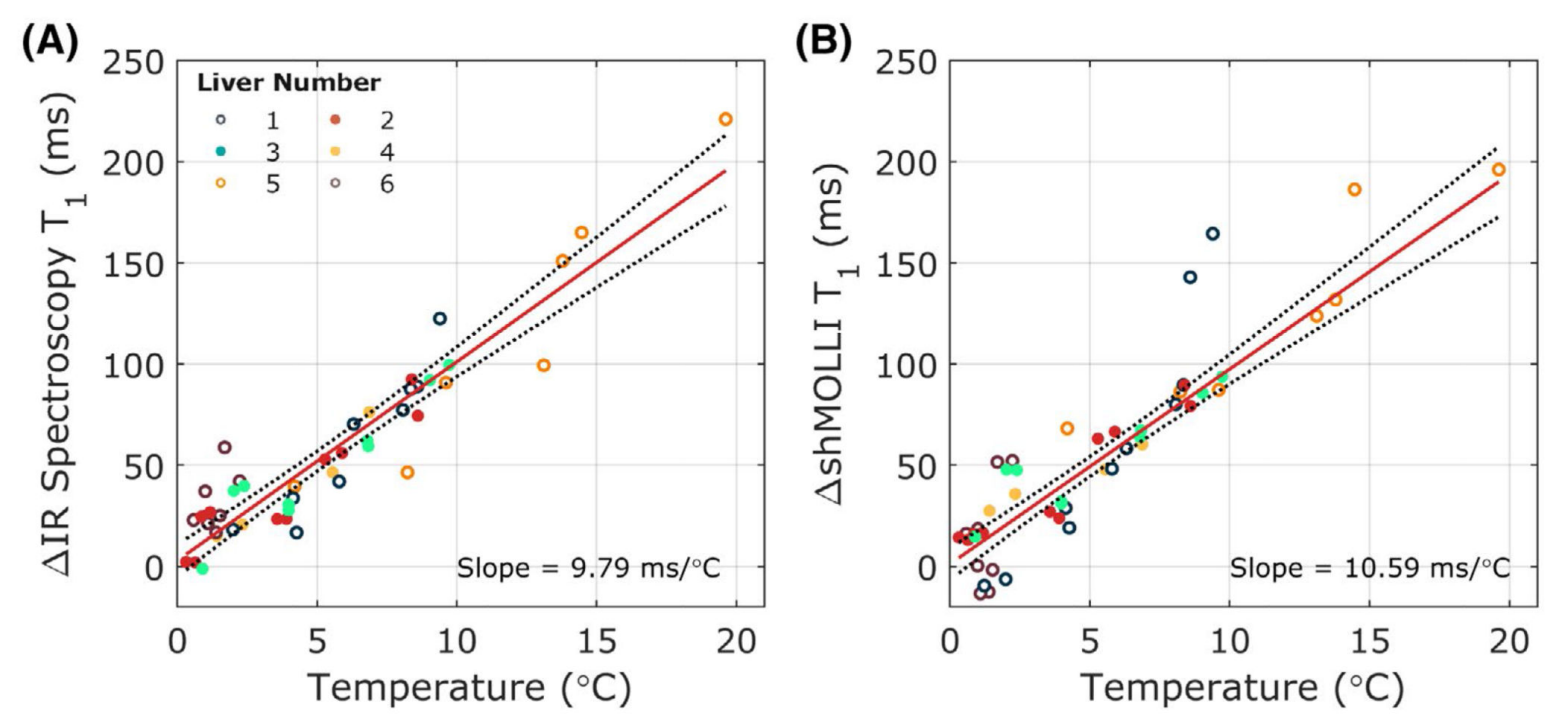
Variation of T_1_ as a function of temperature during static cold storage measured using single voxel STEAM inversion recovery spectroscopy as gold standard (A) and shMOLLI (B) sequences. ShMOLLI T_1_ measurements were calculated from a ROI that matched the spectroscopy voxel position in the center of the right lobe of the liver away from any obvious vasculature. Abbreviations: ROI, region of interest; shMOLLI, shortened MOLLI

A strong linear correlation (*ρ* > 0.84, R^2^ > 0.78, *P* < .0001) was observed between water T_1_ measurements and temperature, with sensitivities of 9.79 ms/°C (7.16-12.43) and 10.16 ms/°C (8.70-11.61) for STEAM-IR T_1_ and ShMOLLI T_1_, respectively, as shown in Figure 3. Application of these temperature sensitivities to calculate temperature-corrected T_1_ values reduced the coefficients of variation from 5.25% and 5.46% to 2.87% and 3.68%, respectively, for the spectroscopic and imaging techniques, as shown in Table 2. Similar decreases were observed in the coefficients of repeatability following temperature correction from 110 ms and 98 ms to 58 ms and 63 ms for STEAM-IR T_1_ and ShMOLLI T_1_, respectively.

**Figure 4:**
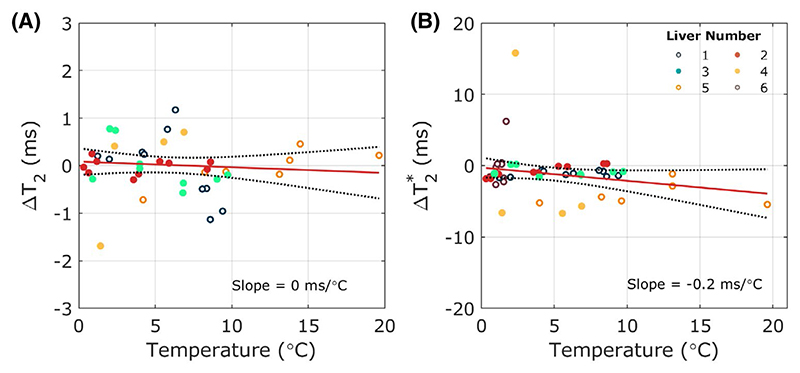
(A) Variation of T_2_ as a function of temperature during static cold storage measured using a multi-TR, multi-TE single voxel STEAM spectroscopy sequence. (B) Variation of ${\text{T}}_{2}^{*}$ as a function of temperature in the same livers. ${\text{T}}_{2}^{*}$ was measured using a multiple-echo spoiled gradient-recalled echo sequence with IDEAL reconstruction. ${\text{T}}_{2}^{*}$ was calculated from a ROI that matched the position of the spectroscopy voxel in the center of the right lobe of the liver away from any obvious vasculature. Abbreviation: IDEAL, iterative decomposition of water and fat with echo asymmetry and least-squares estimation

No significant correlations were observed between water T_2_ measured using multi-TR, multi-TE spectroscopy or ${\text{T}}_{2}^{*}$ measured using a multi-echo spoiled gradient echo imaging sequence and temperature (Figure 4). However, repeated measurements within a liver were very reproducible with coefficients of repeatability of 1.32 ms for T_2_ measurements and 10.23 ms for ${\text{T}}_{2}^{*}$ measurements. Variation between livers was larger with values of 25.9 ± 14.7 ms and 20.7 ± 7.6 ms seen across all livers for T_2_ and ${\text{T}}_{2}^{*}$ respectively. Liver 6 was excluded from analysis of multi-TR multi-TE spectroscopy data as multi-TR; multi-TE spectroscopy data were not acquired. Only 5 and 6 measurement timepoints of all MR parameters were acquired for livers 4 and 5 respectively.

**Figure 5:**
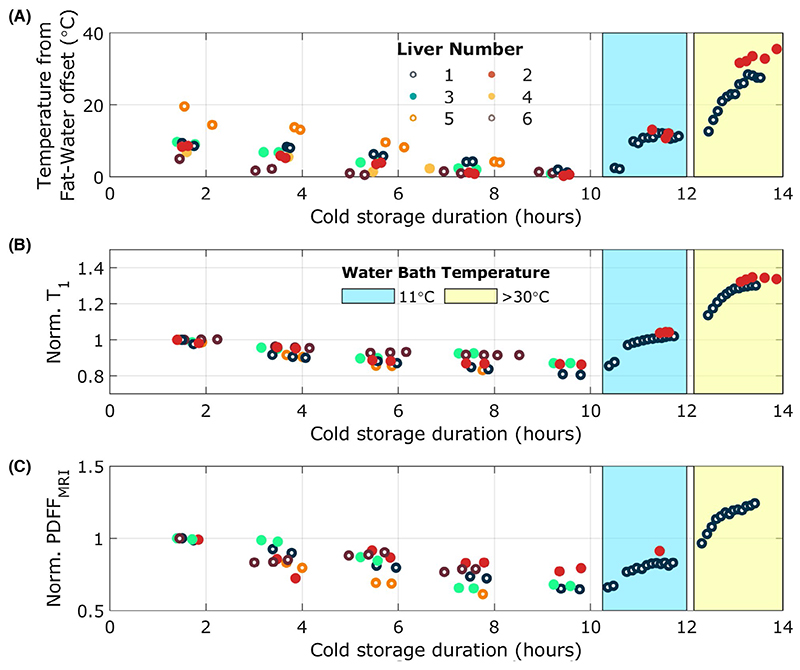
Changes in temperature estimated from the fat–water offset frequency (A), ShMOLLI T_1_ (B), and MRI PDFF (C) as a function of time. ShMOLLI T_1_ and MRI PDFF are both normalized to the initial value. Following 10 h of static cold storage, 2 livers were placed into a water bath at 11°C for 90 min before being placed into a water bath at 30°C (liver 1) or 35°C (liver 2) to re-warm. Abbreviations: PDFF, proton density fat fraction; ShMOLLI, shortened MOLLI

When Livers 1 and 2 were re-warmed using a water bath, the T_1_ values returned to within 3% of the initial value having been held at the starting temperature of 11°C for 90 min and then proceeded to increase further following immersion in a water bath at 30°C (liver 1) and 35°C (liver 2) for 90 min, as shown for ShMOLLI T_1_ in Figure 5B. For data recorded during immersion in the second (warm) water bath, calculated T_1_ and temperature values were still increasing when the experiment was stopped, suggesting that thermal equilibrium was not reached.

### 3.4 Quantification of steatosis

**Figure 6:**
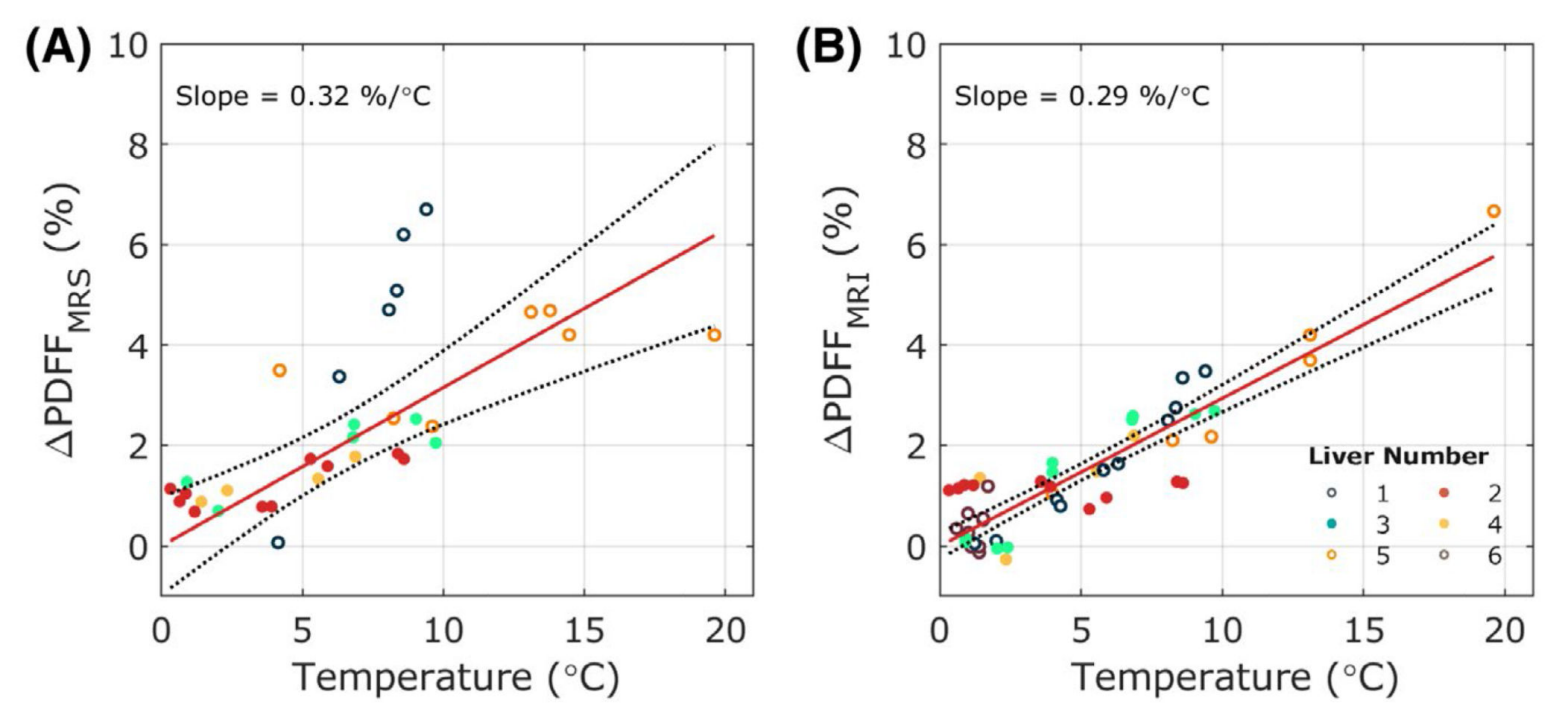
Variation of calculated PDFF as a function of temperature for a T_1_- and T_2-_corrected multi-TR multi-TE STEAM single voxel spectroscopy sequence (A) and an IDEAL multi-echo imaging sequence (B). Imaging-based PDFF measurements were calculated from a ROI that matched the spectroscopy voxel position in the center of the right lobe of the liver away from any obvious vasculature. Abbreviations: IDEAL, iterative decomposition of water and fat with echo asymmetry and least-squares estimation; PDFF, proton density fat fraction

Figure 6 shows the correlation between PDFF and temperature (*ρ* = 0.65 and 0.78, R^2^ = 0.42 and 0.61 for spectroscopic and imaging measurements, respectively, *P* < .001). The 2 PDFF measures, calculated from spectroscopic and imaging data respectively, were only slightly sensitive to temperature with slopes of 0.22%/°C (0.10-0.33) and 0.26%/°C (0.21-0.30). As with T_1_ data, performing a temperature correction reduced the intra-liver measurement variation, decreasing the coefficient of repeatability from 2.40% and 1.08% to 2.08% and 0.64% spectroscopic and imaging-based PDFF, respectively (Table 2).

The coefficient of variation increased from 27.55% to 36.05% for spectroscopic PDFF and decreased from 22.67% to 17.42% for the MRI PDFF following temperature correction.

During re-warming to 11°C, the measured fat fraction increased but only to 85% of the first measurement during SCS (Figure 5C). Increasing the water bath temperature (to 30°C and 35°C for liver 1 and 2, respectively) saw further increases in measured fat fraction up to a maximum of 128% of the first fat fraction measurement. However, as with calculated temperature and T_1_ values, the calculated fat fraction was still increasing when the experiment was stopped, suggesting that thermal equilibrium was not reached.

### 3.5 Correlation of imaging parameters with histology and liver function

**Figure 7:**
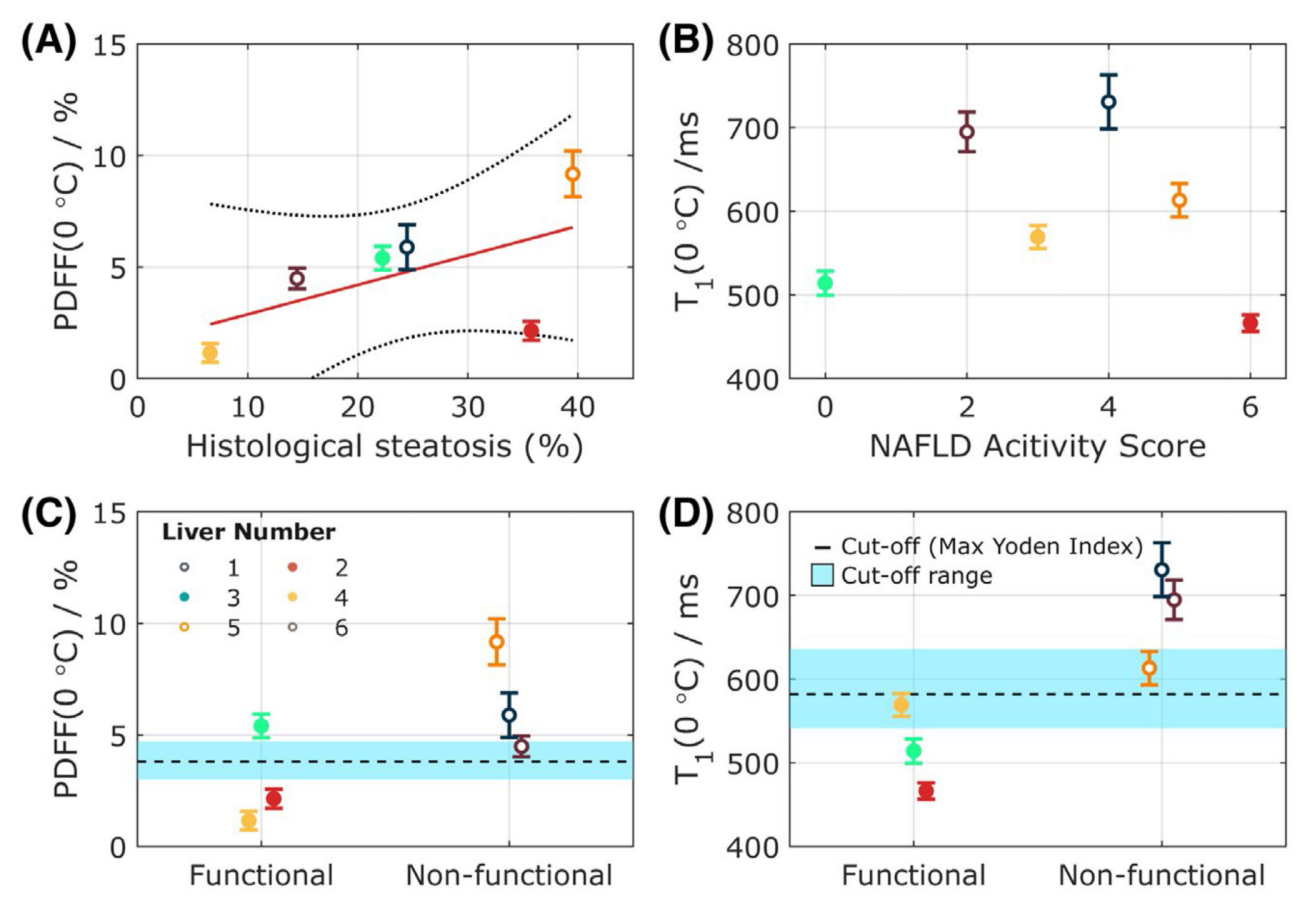
Correlations of PDFF and T_1_ with histological and functional assessment biomarkers in ex vivo human livers. (A) Correlation of temperature-corrected PDFF, PDFF (0°C), with macrovesicular steatosis measured using biopsy samples. (B) A positive but nonsignificant correlation of temperature-corrected ShMOLLI T_1_, T_1_(0°C), with NAFLD activity score. (C) and (D) show differences in PDFF (0°C) and ShMOLLI T_1_(0°C) between livers deemed functional during normothermic machine perfusion by viability criteria and those deemed nonfunctional. Only the difference in T_1_ is statistically significant (*P* < .001). The range of possible cutoff values for classifying functional versus nonfunctional livers are indicated. The dashed line shows the cutoff values with the maximum Yoden Index. The blue shaded region is the range of cutoffs achieving > 80% sensitivity and specificity in our study. Further work will be needed to recommend and validate a specific cutoff value for clinical studies. Abbreviations: NAFLD, nonalcoholic fatty liver disease; PDFF, proton density fat fraction; ShMOLLI, shortened MOLLI

Figure 7A shows the significant correlation of temperature-corrected PDFF with macrovesicular steatosis content measured in biopsy samples (*ρ* = 0.57, R^2^ = 0.41, *P* < .05). A similar correlation was observed for spectroscopic PDFF (Supporting Information Figure S1). Correlations of ShMOLLI T_1_ and STEAM-IR T_1_ values with nonalcoholic fatty liver disease activity score measured from biopsies were all positive but not statistically significant (Supporting Information Figure S2).

Significant differences were observed in both imaging (ShMOLLI) and spectroscopic (STEAM-IR) T_1_ measurements between the livers deemed functional by the viability criteria and those deemed nonfunctional (516 ± 52 ms vs. 679 ± 60 ms, *P* = .02, for ShMOLLI T_1_; 636 ± 50 ms vs. 762 ± 25 ms, *P* = .02, for STEAM-IR T_1_). Livers deemed functional also had a trend toward lower PDFF than livers deemed nonfunctional, but this trend was not significant (2.9 ± 2.2% vs 6.5 ± 2.4, *P* = .13, using MRI PDFF). A range of cutoff values for T_1_ and PDFF would separate functional from nonfunctional livers with sensitivity and specificity > 80% in the small group of livers assessed in this study (see Figure 7 and Supporting Information Table S3).

## 4 Discussion

This study demonstrates that temperature is a confounding factor for MRI- and MRS-based T_1_ and PDFF measurements in ex vivo human livers during SCS. Hence, we propose a correction enabling the estimation of temperature independent values. Temperature-corrected values of fat fraction and T_1_ have shown promise for noninvasive assessment of macrovesicular steatosis level and prediction of liver function during normothermic machine perfusion.

The temperature changes observed in this study (more than 9°C) are consistent with previous invasive temperature measurements of livers during SCS^17,36^ and highlight the need to account for temperature effects in any proposed biomarkers. The fat–water frequency offset provides a reliable noninvasive temperature measurement in ex vivo human livers with an average error of ±1.13°C compared to fiber optic temperature probes as the gold standard. This error is only slightly more than the reported ±0.8°C uncertainty of the fiber optic temperature probes themselves.^37^ Some of the additional variation is likely due to spatial variation in liver temperature because we used an average of multiple voxels for a global assessment of liver temperature. The temperature sensitivity of fat–water frequency offset in ex vivo human livers flushed with HTK preservation solution is very similar to reported values in phantoms^13^ and a range of animal tissues.^22^ This suggests that, although we have validated this approach here only for ex vivo human livers, it also is likely to be more widely applicable to studies of other ex vivo human organs.

Monitoring the same organs over time and fitting measured values to a linear mixed model enables the background variation due to underlying pathological variability between livers to be removed and provides tight estimates of the temperature sensitivities of the MR parameters studied. All of the T_1_ quantification methods used showed very similar temperature sensitivities, suggesting that the other confounding factors that affect ShMOLLI T_1_ measurements but have less impact on spectroscopic inversion recovery T_1_ measurements (eg, iron concentrations^38^) are independent confounders whose influence may not change so much with temperature. It should be noted, however, that these confounding factors are still present; therefore, each of the different measurement techniques calculated different T_1_ values with different ranges of possible cutoff values.

The temperature sensitivity of T_1_ values reported in this study is comparable to the temperature sensitivity of cadaveric human livers as reported by Zech et al., even though blood was present in cadavers and was replaced with HTK preservation solution in the present study.^15^ In the study, Zech et al. measured T_1_ using an inversion recovery sequence in cadavers with a temperature sensitivity of 11.026 ms/°C.^15^ The authors also noted very little or no temperature sensitivity of T_2_ in cadaveric livers, which is consistent with our findings in ex vivo human livers.

Significant linear correlations were also seen for the variation of PDFF measurements with temperature (*ρ* = 0.65, R^2^ = 0.42 for spectroscopic PDFF measurements and *ρ* = 0.77, R^2^ = 0.60 imaging-based PDFF measurements, both *P* < .001). Spectroscopic and imaging-based PDFF methods had similar small temperature sensitivities, indicating that performing a T_1_ and T_2_ correction to the raw spectroscopy data^23^ or a frequency correction to the simulated fat spectrum for the imaging data^13^ substantially compensates for variation in the measurement due to temperature-induced changes in tissue relaxation parameters. Navaratna et al. have recently demonstrated a similar reduction of temperature sensitivity for MRI PDFF measurements through improved fat spectrum frequency correction in phantom experiments.^39^ We believe that much of the remaining variation in measured PDFF could be due to a partial change of state in the fat; that is, we suggest that some hepatic macrovesicular fat droplets begin to solidify at cold temperatures and become “MR-invisible” due to the extremely short T_2_ values seen in this semi-solid “frozen droplet” state. This would accord with results from food science literature, where increased solid fat content, measured by increased contribution of an ultrashort T_2_ compartment, has been seen at lower temperatures in a wide range of sub-stances from lard to chocolate.^40,41^

The recovery of T_1_ values following re-warming to 11°C in a water bath almost to their initial values, as well as the increased values following further warming, indicate that the trends observed in this study are a true function of temperature rather than preservation time. Additionally, the slower recovery of the measured fat concentration could support our suggestion of a partial state change, which could cause a delayed response on warming due to temperature hysteresis effects caused by the rapid re-warming that we employed.

Placing the livers on normothermic machine perfusion before the MR examination provided a method for standardizing the initiation of SCS and an opportunity to observe the livers during very short durations of SCS, which is not possible following the initial retrieval due to the liver sample being transported to the MR center from the donor hospital — a process that took a minimum of 9 h in this study. Additionally, the functional data from a liver during NMP acted as a surrogate endpoint to enable a preliminary assessment of the potential for our MRI and MRS approach to predict viability for transplantation. The viability criteria we used to determine if a liver is functional, and hence viable for transplantation, have been previously used to safely transplant over 20 livers previously declined for transplantation with 100% 90-day graft survival.^9^ Hence, these criteria make a good surrogate marker for a liver’s viability for transplantation. However, it should be noted that the negative predictive value of the current cut-offs used is not known for human livers because the current limits are set conservatively to prevent posttransplant complications. Perfusion was performed for 48 h in the current study which, although not performed clinically, has been shown to keep a liver viable in previous preclinical porcine studies that safely transplanted livers following 48 h of NMP.^42^

Utilizing the liver’s function during NMP as a surrogate marker for transplant viability, we have shown stark differences in T_1_ between functional and nonfunctional livers (516 ± 50 ms vs. 679 ± 60 ms, *P* = .02 for ShMOLLI T_1_ measurements), which warrants further studies into the predictive power of T_1_ relaxation rate of liver grafts for transplantation.

However, our work is a hypothesis-generating study, limited by the number of livers that we could scan and the reliance on a surrogate marker of viability for transplantation instead of scanning during the clinical transplant pathway itself and assessing patient outcomes. We now suggest that a larger scale prospective study scanning livers before transplantation is needed to ascertain whether any of the MR parameters reported here are ultimately predictive of post-transplant outcome. Additionally, all the livers in this study were scanned following NMP, making it impossible to be certain whether the techniques would be predictive in a liver preserved solely by SCS. Finally, ShMOLLI T_1_ measurements in the liver can be corrected for confounding factors such as fat and iron concentrations.^43^ These corrections were not performed in this study. However, our data suggest that T_2_ and ${\text{T}}_{2}^{*}$ show little variation with temperature; hence, with a larger sample size it should in future be possible to update the model proposed by Mozes et al. for ex vivo applications.^43^

In conclusion, we have characterized the temperature sensitivities of tissue relaxation parameters and proton density fat fraction in ex vivo cold-stored human livers measured using both MRI and MRS. This enables temperature correction of these parameters. Our findings in 6 livers using NMP performance as a proxy for transplant viability suggest that this approach has potential to predict the viability of donated livers for transplantation and is worth further study in a suitably powered prospective study.

## Supplementary Material

SI

## Figures and Tables

**Table 1:** Donor demographics, parameters characterising liver function during normothermic machine perfusion and histology results for cold-stored discarded human livers

Liver Number	1	2	3	4	5	6
Donor characteristics						
Age (y)	44	40	72	30	61	44
Sex	M	M	F	M	M	F
Type	DCD	DBD	DBD	DBD	DCD	DCD
Cause of death	HBI	MI	HBI	ICH	TBI	ICH
Cold ischemia time (min)	730	663	899	546	689	1122
NMP duration (h)	48	48	48	48	36	48
Histological steatosis (%)	24.47	35.76	22.26	6.61	39.53	14.49
Liver function during normothermic machine perfusion						
[Lactate]_(4 h)_ (mmol L ^–1^)	2.4*	2.4*	1.0*	2.5*	4.0	2.3*
[Lactate]_(end)_ (mmol L^–1^)	4.2	1.6*	1.9*	1.4*	14.2	5.6
pH_(48 h)_	7.28	7.20	7.24	7.20	6.93	7.34*
Mean hepatic artery flow rate (dm^3^ min^–1^)	0.51 ± 0.03*	0.56 ± 0.05*	0.42 ± 0.09*	0.33 ± 0.11*	0.38 ± 0.08*	0.50 ± 0.08*
Mean portal vein flow rate (dm^3^ min^–1^)	1.04 ± 0.03*	1.05 ± 0.06*	1.01 ± 0.03*	1.17 ± 0.06*	1.04 ± 0.08*	1.11 ± 0.06*
Total bile production (mL)	360*	0	80*	400*	320*	0
Maximum glucose metabolism (mmol/L/h)	–3.1*	–3.3*	–4.93*	–3.1*	–2.3*	–3.5*
Homogeneous perfusion	Yes	Yes	Yes	Yes	No	Yes
Viability criteria met:	No	Yes	Yes	Yes	No	No

*Note:* Perfusion parameters meeting the viability criteria for transplant viability are signified by an asterisk. Abbreviations: DBD, donor after brain-stem death; DCD, donor after circulatory death; F, female; HBI, hypoxic brain injury; ICH, intracerebral hemorrhage; M, male; MI, myocardial infarction; NMP, normothermic machine perfusion; TBI, traumatic brain injury.

**Table 2:** Temperature sensitivities of T_1_, T_2_, ${\text{T}}_{2}^{*}$, and PDFF in ex vivo human livers during static cold storage

			Coefficient of Variation (%)		Coefficient of Repeatability (ms)	
	Grahient (ms/°C)	*P* value	Raw data	Temperature-corrected	Raw data	Temperature-corrected
T_1_						
STEAM-IR spectroscopy	9.79 (7.16-12.43)	<.0001	5.25	2.87	110	58
ShMOLLI	10.16 (8.70-11.61)	<.0001	5.46	3.68	98	63
T_2_						
Multi-TR multi-TE spectroscopy	0.00 (–0.05-0.05)	.86	1.72	–	1.32	–
${\text{T}}_{2}^{*}$						
Multi-echo SGRE	–0.12 (–0.35-0.11)	.31	16.20	–	10.59	–
			**Coefficient of variation (%)**		**Coefficient of repeatability (%)**	
	**Gradient (%/°C)**	***P* value**	**Raw data**	**Temperature-corrected**	**Raw data**	**Temperature-corrected**
PDFF_MRS_	0.22 (0.10-0.33)	<.001	27.55	36.05	2.40	2.08
PDFF_MRI_	0.26 (0.21-0.30)	<.0001	22.67	17.42	1.08	0.64

*Note:* The average coefficients of variation and repeatability for repeated measurements in the livers before and, where a significant correlation with temperature is observed, after temperature correction are also presented. Abbreviations: PDFF, proton density fat fraction; SGRE, spoiled gradient echo; STEAM-IR, single voxel inversion recovery STEAM.
