# Supplementary material for: Noninvasive assessment of steatosis and viability of cold-stored human liver grafts by MRI
Source: Magn Reson Med. Author manuscript; Available in PMC 2022 Jul 29. (PMC7613197; doi:10.1002/mrm.28930)
Supplement: SI [file EMS150772-supplement-SI.docx]

Supplementary Material

Non-invasive assessment of steatosis and viability of cold stored human liver grafts by MRI

**Table S1** Fat spectrum model used for IDEAL reconstructions. The frequencies of the fat peaks modelled are adjusted by ß ppm per degree C, where ß = 0.01 as reported by Hernando et al. Multiple-TR multiple-TE spectroscopy used peaks 1, 2 and 3 in the prior knowledge file for fitting and then accounted for the other peaks in the spectrum by weighting calculated fat signal by the relative proportion of the fat spectrum signal accounted for by the peaks fitted. The single-TE spectroscopy used all fat peaks in the prior knowledge of the water suppressed spectrum.

| Peak Number | Species | Chemical Shift (ppm) | Relative Amplitude (a.u.) |
| --- | --- | --- | --- |
| Fat spectrum |  |  |  |
| 1 | Methyl | 0.90 – β × ΔT | 0.087 |
| 2 | Methylene | 1.30 – β × ΔT | 0.693 |
| 3 | α-carbonyl and α-olefinic | 2.10 – β × ΔT | 0.128 |
| 4 | Diacyl | 2.76 – β × ΔT | 0.004 |
| 5 | Glycerol | 4.30 – β × ΔT | 0.039 |
| 6 | Olefinic | 5.30 – β × ΔT | 0.049 |
| Water spectrum |  |  |  |
| 7 | Water | 4.70 | 1 |

**Table S2.** Temperature sensitivities of T_1_, T_2_, T_2_*, fat fraction and proton density fat fraction in ex vivo human livers during static cold storage. Additionally, the average coefficients of variation and reproducibility for repeated measurements in the livers before and, where a significant correlation with temperature is observed, after temperature correction. Spectroscopic fat fraction (FF_MRS_) was calculated as the ratio of the total fat signal from the six peaks in the water supressed single-TE STEAM spectra and the sum of the total fat signal in the water supressed spectrum plus the water peak amplitude in the reference spectrum acquired without water suppression.

|  | **Gradient**  **(ms / °C)** | **p-value** | **Coefficient of variation (%)** | | **Coefficient of repeatability (ms)** | |
| --- | --- | --- | --- | --- | --- | --- |
|  |  |  | **Raw data** | **Temperature corrected** | **Raw data** | **Temperature corrected** |
| **T_1_** | | | | | | |
| STEAM-IR Spectroscopy | 9.79 (7.16–12.43) | <0.0001 | 5.25 | 2.87 | 110 | 58 |
| Multiple-TR multiple-TE Spectroscopy | 10.48 (8.23–12.74) | <0.0001 | 6.34 | 3.54 | 112 | 60 |
| ShMOLLI | 10.16 (8.70–11.61) | <0.0001 | 5.46 | 3.68 | 98 | 63 |
| **T_2_** | | | | | | |
| Multi-TR multi-TE Spectroscopy | 0.00 (-0.05-0.05) | 0.86 | 1.72 | - | 1.32 | - |
| **T_2_*** | | | | | | |
| Multi-echo GRE | -0.12 (-0.35 – 0.11) | 0.31 | 16.20 | - | 10.59 | - |
|  | **Gradient**  **(% / °C)** | **p-value** | **Coefficient of variation (%)** | | **Coefficient of repeatability (%)** | |
|  |  |  | **Raw data** | **Temperature corrected** | **Raw data** | **Temperature corrected** |
| FF_MRS_^†^ | 0.47 (0.40 – 0.54) | <0.0001 | 27.22 | 32.78 | 5.19 | 3.50 |
| PDFF_MRS_ | 0.22 (0.10-0.33) | <0.001 | 27.55 | 36.05 | 2.40 | 2.08 |
| PDFF_MRI_ | 0.26 (0.21-0.30) | <0.0001 | 22.67 | 17.42 | 1.08 | 0.64 |
| ^†^ *FF_MRS_ was calculated from water suppressed and unsuppressed spectra which had different contrasts (repetition times). Therefore, there may be an additional bias present in these measurements.* | | | | | | |


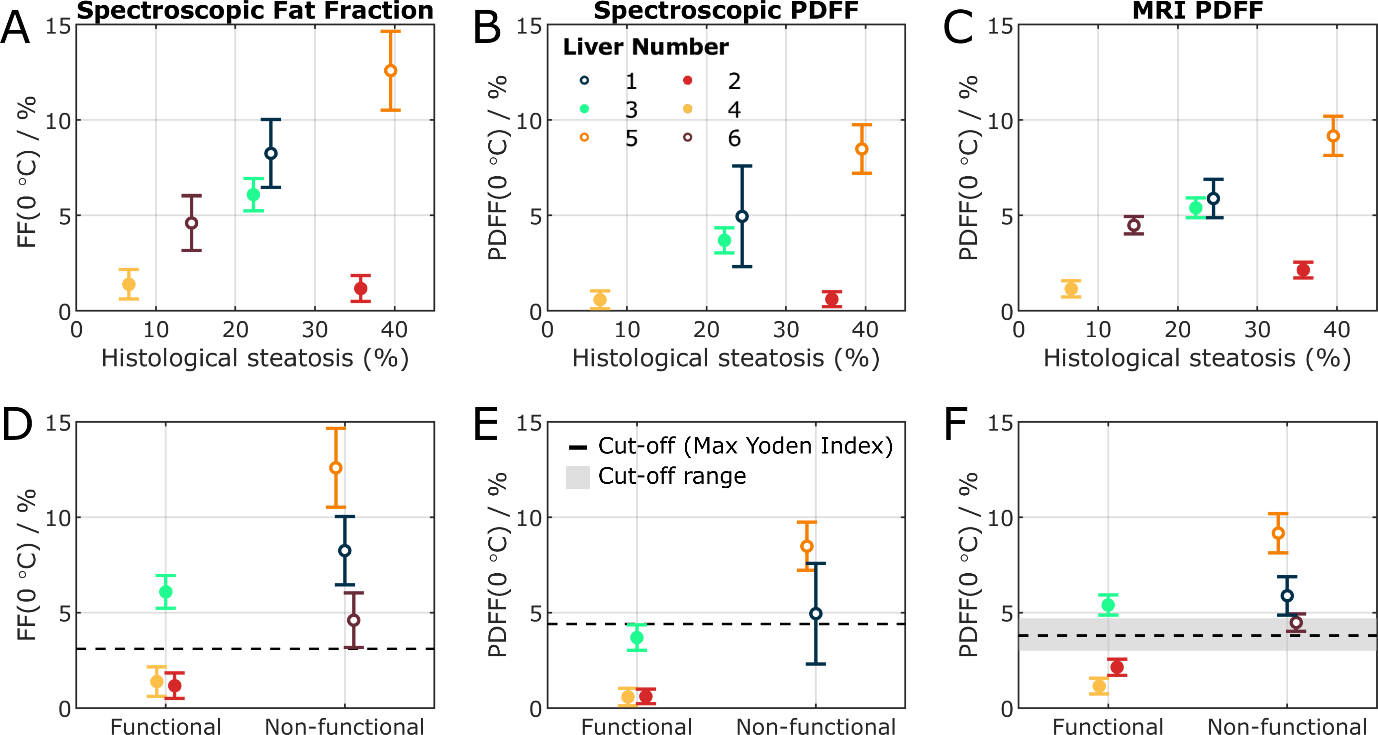


**Figure S1** Correlations of fat fraction and proton density fat fraction with histological and functional assessment biomarkers in ex vivo human livers. (A) Correlation of temperature corrected spectroscopic fat fraction, FF(0°C), with macrovesicular steatosis measured from biopsy samples using ImageJ. (B) and (C) show the same correlations for temperature corrected proton density fat fraction, PDFF(0°C), acquired using spectroscopy and imaging methods respectively. (D - F) show FF(0°C) and PDFF(0°C) values for livers deemed functional during normothermic machine perfusion using the viability criteria and those deemed non-functional. Possible cut-off values for diagnosing non-functional livers are presented at the maximum Yoden Index and as a range which all achieve >80% sensitivity and specificity.


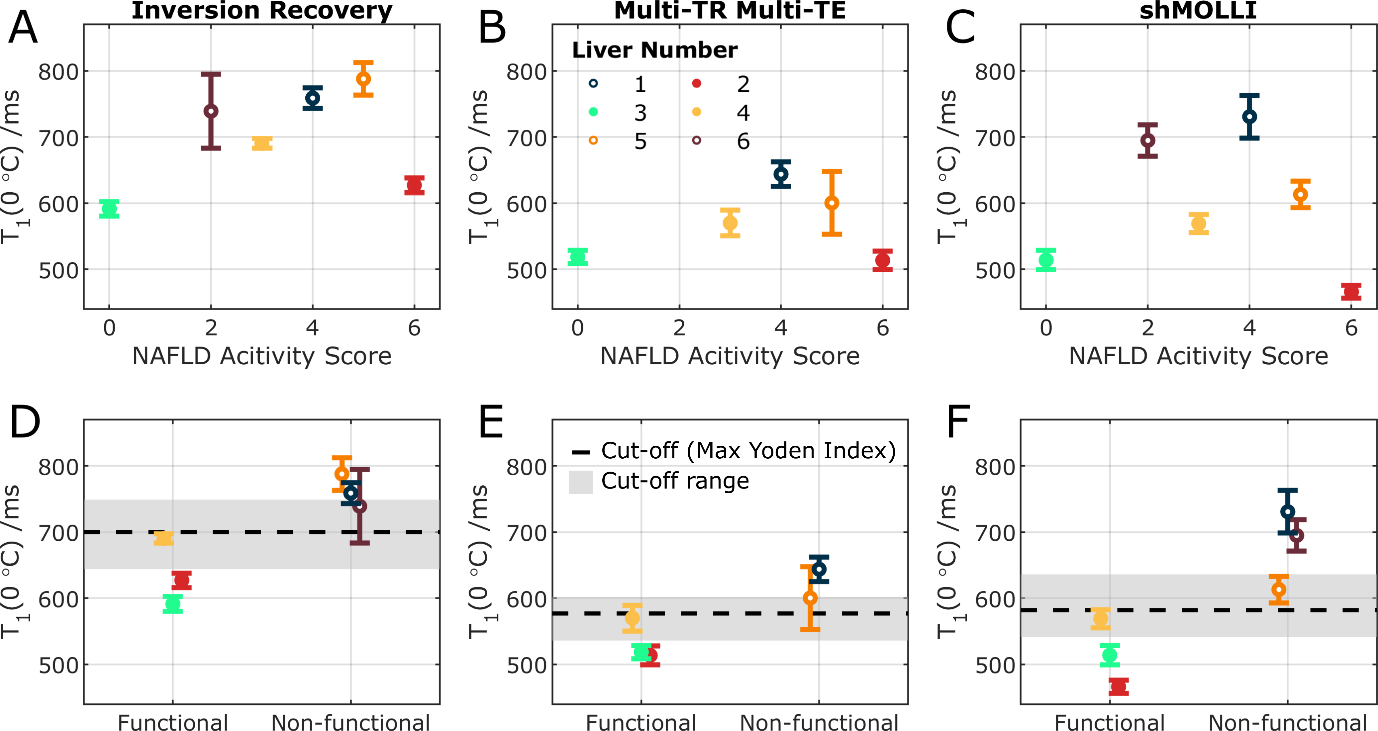


**Figure S2** Correlations of three different measures of T_1_ with histological and functional assessment biomarkers in ex vivo human livers. (A) A positive but non-significant correlation of spectroscopic inversion recovery T_1_ with Non-alcoholic fatty liver disease (NAFLD) activity score. (B) and (C) show similar correlations of NAFLD activity score and T_1_ measured using multi-TR multi-TE spectroscopy and ShMOLLI respectively. (D-F) show differences in T_1_, measured using the same three techniques, between livers deemed functional during normothermic machine perfusion using the viability criteria and those deemed non-functional. Significant differences were observed in both inversion recovery T_1_ and ShMOLLI T_1_ values between functional and non-functional livers (*P* < 0.002). Possible cut-off values for diagnosing non-functional livers are presented at the maximum Yoden Index and as a range which all achieve >80% sensitivity and specificity. Differences in T_1_ measured using multi-TR, multi-TE spectroscopy between functional and non-functional livers were not assessed for statistical significance due to the lack of data for Liver 6.

**Table S3** Possible cut off values for temperature corrected T_1_ and fat fraction measured in static cold stored ex vivo human livers for differentiating between functional and non-functional livers. The cut-off value which maximises the Youden index is presented, together with the range of cut-off values that achieve sensitivity and specificity >80%. Further work will be needed to recommend and validate a specific cut-off for clinical studies.

|  | **Cut-off value at maximum Youden Index (ms)** | **Maximum Youden Index** | **Range of cut-off values (ms)** |
| --- | --- | --- | --- |
| IR Spectroscopy T_1_ | 700 | 0.96 | 644 – 749 |
| Multi-TR multi-TE spectroscopy T_1_ | 577 | 0.86 | 536 – 602 |
| ShMOLLI T_1_ | 582 | 1.00 | 541 – 636 |
|  | **Cut-off value at maximum Youden Index (%)** | **Maximum Youden Index** | **Range of cut-off values (%)** |
| FF_MRS_ | 3.2 | 0.25 | – |
| PDFF_MRS_ | 4.4 | 0.71 | – |
| PDFF_MRI_ | 3.8 | 0.84 | 3.0 – 4.7 |
